# Alterations of the endocannabinoid system and its therapeutic potential in autism spectrum disorder

**DOI:** 10.1098/rsob.200306

**Published:** 2021-02-03

**Authors:** Mingyang Zou, Yu Liu, Shu Xie, Luxi Wang, Dexin Li, Ling Li, Feng Wang, Yujue Zhang, Wei Xia, Caihong Sun, Lijie Wu

**Affiliations:** ^1^ Department of Children's and Adolescent Health, Public Health College of Harbin Medical University, Harbin 150081, People's Republic of China; ^2^ Department of Children Psychology, Zhuhai Maternal and Child Health Care Hospital, Zhuhai 519001, People's Republic of China; ^3^ Office of Leading Group for Control and Prevention of Major Diseases and Infectious diseases, Dezhou Center for Disease Control and Prevention, Dezhou 253011, People's Republic of China

**Keywords:** autism spectrum disorder, endocannabinoid system, VPA-induced rat, JZL184

## Abstract

Autism spectrum disorder (ASD) is a group of developmental disabilities, the aetiology of which remains elusive. The endocannabinoid (eCB) system modulates neurotransmission and neuronal plasticity. Evidence points to the involvement of this neuromodulatory system in the pathophysiology of ASD. We investigated whether there is a disruption to the eCB system in ASD and whether pharmacological modulation of the eCB system might offer therapeutic potential. We examined three major components of the eCB system—endogenous cannabinoids, their receptors and associated enzymes—in ASD children as well as in the valproic acid (VPA) induced animal model in autism. Furthermore, we specifically increased 2-arachidonoylglycerol (2-AG) levels by administering JZL184, a selective inhibitor of monoacylglycerol lipase which is the hydrolytic enzyme for 2-AG, to examine ASD-like behaviours in VPA-induced rats. Results showed that autistic children and VPA-induced rats exhibited reduced eCB content, increased degradation of enzymes and upregulation of CBRs. We found that repetitive and stereotypical behaviours, hyperactivity, sociability, social preference and cognitive functioning improved after acute and chronic JZL184 treatment. The major efficacy of JZL184 was observed after administration of a dosage regimen of 3 mg kg^−1^, which affected both the eCB system and ASD-like behaviours. In conclusion, a reduced eCB signalling was observed in autistic children and in the ASD animal model, and boosting 2-AG could ameliorate ASD-like phenotypes in animals. Collectively, the results suggested a novel approach to ASD treatment.

## Graphical abstract

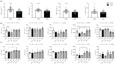

## 1. Introduction

Autism spectrum disorder (ASD) is a collection of heterogeneous neurodevelopmental disorders and it is defined by impairment in communication and social interactions, as well as restricted, repetitive patterns of behaviour [1]. ASD affects approximately 1% of children in mainland China [2], which is comparable to Western countries, and its prevalence seems to be cumulatively increasing. The most recent prevalence estimate of ASD reached 1.85% (one in 54) among children aged 8 years old [3]. Despite its high prevalence and the public health burdens that result, there is a relatively limited understanding of the pathophysiology of ASD, aside from complex interactions between genetic and environmental factors. A multitude of recent publications have suggested that ASD is related to abnormalities in synaptic function. Thus, the endocannabinoid (eCB) system has attracted increasing interest for its potential in the onset and/or progression of ASD, as this system could modulate different neurotransmitter system, synaptic excitation and inhibition (E/I balance) and plasticity in the brain, and it could also be associated with social interaction, motor control, repetitive behaviours, emotional processing, learning and memory.

The eCB system consists of three major components, i.e. endogenous cannabinoids (eCBs), their receptors and associated enzymes. The most active eCBs are anandamide (AEA) and 2-arachidonoylglycerol (2-AG), which act mainly through cannabinoid type-1 and type-2 receptors (CB1Rs and CB2Rs) that are distributed throughout the central nervous system (CNS). In addition, palmitoylethanolamide (PEA) and oleoylethanolamide (OEA), which are structurally similar to AEA (collectively known as *N*-acylethanolamines), share the same catalyzed enzymes required for their metabolism. *N*-acylphosphatidylethanolamine-specific phospholipase D (NAPE-PLD) and diacylglycerol lipase (DAGL), which are involved in the synthesis of *N*-acylethanolamines and 2-AG, respectively. Given that eCBs are not stored in any cellular compartment for later use, they are rapidly inactivated by their hydrolytic enzymes, i.e. fatty acid amide hydrolase (FAAH) and monoacylglycerol lipase (MAGL). MAGL, as the predominantly 2-AG degraded enzyme, accounts for up to 85% of 2-AG hydrolysis in the brain [4]. Inhibitors of FAAH and MAGL are the most common tools to manipulate signalling of eCBs. The eCBs are produced on demand from membrane-bound phospholipids in postsynaptic neuronal membranes and act as retrograde messenger on presynaptic CBRs to dampen the release of neurotransmitters (e.g. monoamine, opioids, GABA, glutamate, acetylcholine), thereby affecting a wide range of biological processes [5]. The eCB-mediated retrograde suppression is considered to be a ubiquitous and important form of activity-dependent synaptic modulation.

Indeed, the dysregulation of the eCB system has been documented in relation to several neuropsychological and neurodevelopmental diseases, including ASD. Two case-control studies found decreased circulating levels of AEA, PEA and OEA in ASD children compared with matched healthy children [6,7]. Siniscalco's team reported alterations of the receptors and enzymes in the eCB system in the peripheral blood mononuclear cells (PBMCs) of autistic children [8,9]. It was also suggested that disruption to eCB metabolism was observed in the brain in the case of both genetic and environmental models of ASD. However, these studies appeared to contradict one another. For instance, in *Fmr1* knockout mice, the most common genetic form of autism, MAGL activation was enhanced in the frontal cortex and striatum [10]. In VPA-induced rats, the well-known environmental-based model, there was a reduction in MAGL expression and unaltered levels of AEA, PEA, OEA and 2-AG [11]. Melancia *et al.* [12] showed that CB1R activation decreased, while Zamberletti *et al.* [13] found CB1R upregulation. BTBR, an inbred mouse strain known to model of idiopathic autism, revealed a higher density of CB1Rs [14]. Furthermore, enhancing AEA signalling partially attenuated social behaviour deficits in these three ASD models [12,15,16]. Although still debated, it is plausible that alternations of the eCB system may contribute to the pathogenesis of ASD.

Generally speaking, 2-AG, which is the most abundant eCB in the brain, is found in much higher concentrations than *N*-acylethanolamines in the brain (i.e. approx. 1000-fold higher than AEA), and it executes full agonist activity at CBRs with a high efficacy, while AEA is a partial agonist, and PEA and OEA have a lower affinity [5]. Considering that 2-AG plays a broader role in the integrity of the brain's eCB system and CNS development, 2-AG may be a more relevant indicator of eCB tone [17]. However, changes in 2-AG levels have not been reported in individuals with ASD, and evidence of whether enhancing 2-AG tone could cause an improvement in ASD-like behaviours is limited [18,19]. In this study, we examined all three major components of the eCB system, namely, eCBs (AEA, PEA, OEA and 2-AG), CBRs (CB1R and CB2R) and related catalyzed enzymes (NAPE-PLD, FAAH, DAGL and MAGL), in ASD children as well as in the VPA-induced ASD animal model, in order to comprehensively characterize the involvement of the eCB system in the pathogenesis of ASD. In addition, we investigated the effect of altered 2-AG signalling on autistic behaviours, and examined whether behavioural changes exhibited by VPA-induced rats are associated with eCB dysfunction in discrete brain regions, which are known to module cognitive and social behaviour, namely the hippocampus and prefrontal cortex (PFC). The present study was designed to better understand the critical role of eCB system in the aetiology of ASD, and provide a novel strategy for the treatment in managing symptoms of ASD.

## 2. Material and methods

### 2.1. Participants

We investigated 70 autistic patients and 70 age- and gender-matched controls (age range 3–12). The 70 autistic patients were recruited from the Child Development and Behaviour Research Center of Harbin Medical University and special education schools, Harbin, China. The inclusion criterion was a diagnosis of ASD, which was made by two independent specialist clinicians according to the *Diagnostic and Statistical Manual of Mental Disorders*, 5th edition (DSM-5) [1]. Exclusion criteria were children with significant sensory and motor impairment, known genetic disorders, seizures at the time of enrolment or other neurological disorders. Seventy unrelated healthy children without a history of developmental delay or other neurological disorders were randomly selected from normal kindergartens and junior schools in Harbin, China as the control group. All procedures are conducted with the written consent of the guardians or parents and approved by the ethics committee of Harbin medical university prior to the study.

The following measures were used as an aid for diagnosis and assessment: Autism Diagnostic Observation Schedule (ADOS), Autism Diagnostic Interview-Revised (ADI-R), Autism Behaviour Checklist (ABC), Childhood Autism Rating Scales (CARS), Vineland Adaptive Behaviour Scale second edition (VABS) and Social Responsiveness Scale (SRS). Sample characteristics are provided in electronic supplementary material, table S1.

### 2.2. Animals and treatments

Adult male and female Wistar rats were purchased from a commercial breeder (YISI, Benxi, China) and housed four per cage in a controlled environment (22°C ± 2°C; 50% ± 10% humidity). All rats had free access to water and food and were maintained on a 12 h light/dark cycle. Animals were allowed to acclimatize for one week prior to the experiments. The rat model of ASD was established according to previous studies [20,21]. Briefly, female and male rats were allowed to mate overnight. Pregnancy was determined by the presence of a vaginal plug the next morning, and noon of that day was defined as the embryonic day (E)0.5. Sodium valproic acid (VPA; Sigma-Aldrich, St Louis, MO, USA) was dissolved in 0.9% saline at a concentration of 250 mg ml^−1^, and pregnant rats received a single intraperitoneal (i.p.) injection of 600 mg kg^−1^ VPA or an equal volume of saline (VPA-treated and control groups, respectively) on E12.5. Pregnant rats were individually housed and allowed to raise their own litters. The offspring were weaned on postnatal day (PND) 21 and housed with 4–5 per cage. Experiments were performed on male offspring.

Animals were administered (4-nitrophenyl) 4-[bis(1,3-benzodioxol-5-yl)-hydroxymethyl] piperidine-1-carboxylate (i.p., JZL184, Selleck, Houston, TX, USA), a selective inhibitor of MAGL that enhances the levels of 2-AG. JZL184 was dissolved in dimethyl sulfoxide (DMSO) to prepare a mother liquor at a concentration of 50 mg ml^−1^. It was then diluted with DMSO (5%), PEG400 (40%), Tween 80 (5%) and double distilled water into a clear working solution made up to a volume of 2.5 mg ml^−1^. Doses of JZL184 were selected based on the results of previous studies [22,23]. The male offspring from different dams were randomly divided into six groups as follows (figure 3):

(i) CON (injection with vehicle solution);
(ii) VPA (injection with vehicle solution);
(iii) VPA + 40AJ (40 mg kg^−1^, i.p., acute JZL184 injection once on PND35);
(iv) VPA + 1RJ (1 mg kg day^−1^, i.p., repeated JZL184 injection from PND21–34);
(v) VPA + 3RJ (3 mg kg day^−1^, i.p., repeated JZL184 injection from PND21–34);
(vi) VPA + 10RJ (10 mg kg day^−1^, i.p., repeated JZL184 injection from PND21–34).

The increase in brain 2-AG levels by JZL184 administration persisted for at least 26 h, indicating that 2-AG could remain elevated throughout the repeated dosing regimen [17]. Twenty-four hours after the last repeated injection and 2 h after the acute injection, animals from each group were anaesthetized with an intraperitoneal injection of 10% chloral hydrate (0.3 ml kg^−1^). The animals were sacrificed by decapitation for liquid chromatography-tandem mass spectrometry (LC-MS/MS), quantitative PCR (qPCR) and western blot assay. The brains were removed and the hippocampus and PFC were dissected out, flash frozen in liquid nitrogen and stored at −80°C. Starting from PND35, a series of behavioural tests were performed. In the present study, the biochemical tests were carried out on rats of untested behavioural experiments. The behavioural testing and biochemical testing were conducted on 5–9 rats for separate experiments.

### 2.3. Behavioural testing

Behavioural testing was captured by video cameras and analysed using the SMART (Spontaneous Motor Activity Recording and Tracking) v. 3.0 software system (Panlab, Barcelona, Spain). The apparatus was cleaned with 0.1% acetic acid between trials to preclude olfactory cues. All behavioural experiments were performed during the light cycle between 09.00 and 17.00, and testing was counterbalanced across treatment groups.

#### 2.3.1. Marble burying test

A clean cage (48 × 35 × 20 cm) was prepared with 5 cm fresh wood chip bedding material. On PND 35, a rat was placed individually in the cage for 15 min for habituation. They were then returned to their home cage. This rat was reintroduced onto the bedding material containing 20 embedded marbles for 30 min, and the number of marbles buried (i.e. covered with wood chip by more than two-thirds volume) was recorded.

#### 2.3.2. Self-grooming test

On PND 35, the rats were placed individually into a white cage (48 × 35 × 20 cm) and allowed to habituate for 5 min. Self-grooming behaviour was recorded for 10 min. A timer was used to assess the cumulative time spent performing self-grooming behaviour, which included paw licking, unilateral and bilateral strokes around the nose, mouth and face, paw movement over the head and behind ears, body fur licking, body scratching with hind paws, tail licking and genital cleaning.

#### 2.3.3. Open field test

On PND 35, the open field was made out of charcoal grey plastic with a top opening. The dimensions of the test box were 45 × 45 × 40 cm. Before the test, the rats were allowed to adapt to the test box for 5 min. Rats were individually placed in the centre to initiate a 10 min test. The total distance moved and resting time of spontaneous activity, which were used as indicators of anxiety-related behaviours, were analysed.

#### 2.3.4. Three-chamber test

On PND 35, the three-chamber test was used to evaluate the social behaviour of rats. Following acclimation (the rats were placed in the central chamber for 5 min, and the rats were allowed to freely access all chambers), two successive tests (i.e. sociability test and social preference test), which were 10 min in duration, respectively, were carried out. Sociability test: Animals were briefly confined to the central chamber while an unfamiliar rat (labelled as stranger 1) was confined in a small wire cage which was placed in one of the outer chambers. An identical empty wire cage was placed in the other chamber. Social preference test: A novel unfamiliar rat (labelled as stranger 2) was then placed in the empty cage. The sociability index was calculated as the ratio of time spent exploring stranger 1 over the empty cage. The social preference index was calculated as the ratio of the time spent exploring stranger 2 over stranger 1. Familiar and unfamiliar rats originated from different home cages and had never been in physical contact with the subject mice or each other.

#### 2.3.5. Morris water maze test

On PND 36–40, the learning and spatial memory capabilities of rats were evaluated using the Morris water maze test. The apparatus consisted of a circular black water tank (180 cm in diameter and 58 cm deep) filled with water (about 42 cm deep) at a temperature of 19–21°C. The apparatus was concealed with black curtains, with extra visual cues inside the curtains. A circular platform (10 cm in diameter) was always fixed at 2 cm below the water surface in the centre of the first quadrant of the pool. The test period was divided into two phases. Phase 1: The training trial was carried out continuously for 4 days, twice daily in the same time period. The rats were placed into the water facing the tank wall in a set of semi-randomly selected distal starting positions each day, and the escape latency from the start of swimming to reach the platform was recorded as an index of learning. If the rat failed to reach the platform within 60 s and the latency value was recorded as 60 s, then it was guided to the platform and allowed to remain on the platform for 15 s. Phase 2: On the 5th day, the rats were subjected to a spatial probe trial session during which the platform was removed from the pool. The rats entered the pool from the third quadrant, and the number of times that rats passed through the circular area of the original platform within 60 s was recorded as an index of spatial memory. Given that five consecutive days of tests were conducted, this test was not performed with the rats in the acute injection group.

### 2.4. Biochemical testing

#### 2.4.1. Quantitation of eCB levels by LC-MS/MS

Fasting blood samples were collected into EDTA-evacuated tubes in the morning (7.30–8.30) and immediately chilled on ice before centrifuging at 2000 r.p.m. for 20 min at 4°C. Deuterated internal standards AEA-D8, PEA-D4, OEA-D2, 2-AG-D5, arachidonic acid (AA)-D11 (Cayman Chemicals, MI, USA) were used.

A 300 μl volume of methanol (containing internal standards: AEA-D8 at 40 ng ml^−1^, OEA-D2 at 40 ng ml^−1^, PEA-D4 at 40 ng ml^−1^ and 2-AG-D5 at 160 ng ml^−1^) was added to a 100 μl aliquot of plasma sample for protein precipitation. The mixture was eddied for 5 min and centrifuged at 16 000 × *g* for 10 min, and 1 µl of the supernatant was injected into the LC–MS/MS system.

An LC-20ADXR high-performance liquid chromatography (UPLC) system (Shimadzu, Nagoya, Japan) was interfaced with a Sciex Q-trap 5500 mass spectrometer (Applied Biosystems, Foster City, CA, USA) with an electrospray ionization (ESI) source. Data were acquired using Analyst software (v. 1.6.2, Applied Biosystems). The sample vials were maintained at 4°C in a thermostatic autosampler. Chromatographic separation was achieved at 40°C on an Acquity ultra-HPLC HSS T3 column (100 × 2.1 mm, 1.7 µm; Waters, Milford, MA, USA) and VanGuard column (5 mm × 2.1 mm, 1.7 µm; Waters, USA). The mobile phase A was water containing 0.1% formic acid and phase B was acetonitrile. A mobile phase gradient was applied at a flow rate of 0.3 ml min^−1^. The gradient elution was 0–1 min, 5% B; 1–6.0 min, 5–40% B; 6–7 min, 40–100% B; 7–9 min, 100% B. The equilibration time after the gradient was 3 min.

The mass spectrometer was operated in the positive ESI mode with multiple reaction monitoring (MRM) at unit resolution. Nitrogen was used as the nebulizer, heater and curtain gas as well as the collision-activated dissociation gas. The precursor-to-product ion transitions, declustering potential (DP) and collision energy (CE) are listed in electronic supplementary material, table S2. Optimal parameters were as follows: Nebulizer, heater and curtain gas flow rates of 50, 55 and 40 units, respectively; ion spray needle voltage of 5500 V; heater gas temperature of 550°C; and collision gas (N_2_) medium.

A 50 mg (±0.5 mg) section of brain tissue was weighed in a 2.0 ml Lysis Tube containing 1 mm ceramic beads. The sample was homogenized for 60 s by using a Speed Mill PLUS (ANALYTIKJENA). A 100 µl methanol (containing internal standards: AEA-D8, PEA-D4, OEA-D2 and 2-AG-D5 at 100 ng ml^−1^, AA-D11 at 6 µg ml^−1^) and additional 200 µl methanol was added into the sample. The tube was vortexed for 10 s. Then 1 ml MTBE was added and the mixture was incubated for 1 h at room temperature in a shaker. Phase separation was induced by adding 250 µl of MS-grade water. The sample was incubated at room temperature for 10 min and centrifuged at 16 000 × *g* for 10 min. A 500-μl upper (organic) phase was collected and dried in a vacuum centrifuge (Savant SPD131DDA SpeedVac, Thermo fisher). Dry residue was re-dissolved in 200 µl of acetonitrile/isopropanol (1 : 1).

Acquity UPLC H-Class (Waters, Milford, MA, USA) was interfaced to a Waters Xevo tq-s micro mass spectrometer (Milford, Massachusetts, USA) with an ESI source. Data were acquired using Masslyxn (v. 4.1 package, Waters, Milford, MA, USA). The sample vials were maintained at 4°C in a thermostatic autosampler. Chromatographic separation was achieved at 45°C on an Acquity UPLC BEH C8 column (100 mm × 2.1 mm, 1.7 µm; Waters, Milford, MA, USA). The mobile phase A was acetonitrile/water (60/40) and mobile phase B was isopropanol/acetonitrile (90/10). Both A and B contained 0.1% formic acid and 10 mmol l^−1^ ammonium acetate. A mobile phase gradient was applied at a flow rate of 0.3 ml min^−1^. The gradient elution was 0–1 min, 15% B; 1–4.0 min, 15%–40% B; 4–8 min, 40–70% B; 8–9 min, 70–100% B. The equilibration time after the gradient was 3 min.

The mass spectrometer was operated in the positive ESI mode with MRM at unit resolution. Nitrogen was used as the desolvation gas. The precursor-to-product ion transitions, cone voltage and CE are listed in electronic supplementary material, table S2. Optimum parameters were as follows: cone gas flow 10 l h^−1^; capillary voltage 3000 V; desolvation temperature 550°C; desolvation gas flow, 1000 l h^−1^.

#### 2.4.2. Isolation of peripheral blood mononuclear cells

Human venous blood samples from ASD subjects and control donors were drawn and collected in sterile EDTA tubes. Peripheral PBMCs were isolated by centrifugation over Histopaque 1077 density gradient. Briefly, blood was diluted 1 : 1 in phosphate buffer saline (PBS), overlaid onto lymphocyte separation media, centrifuged at 2000 r.p.m. for 30 min at room temperature and plasma was removed. Mononuclear cell fraction was harvested and washed twice in PBS. The final pellet was resuspended in Trizol Reagent (Invitrogen, Thermo Fisher Scientific, Waltham, MA, USA) or protein lysis buffer for further molecular analysis.

#### 2.4.3. RNA extraction, reverse transcription and qPCR

Total RNA was extracted from PBMCs and brain tissue using Trizol Reagent (Invitrogen, Thermo Fisher Scientific, Waltham, MA, USA) according to the manufacturer's protocol. RNA quantity was determined by NanoDrop 2000 spectrophotometer (Thermo Fisher Scientific, Waltham, MA, USA) and purity assessed by A260/A208 ratio. RNA was reverse transcribed to cDNA using high-capacity cDNA reverse transcription kits (Applied Biosystem Inc., Foster City, CA, USA), with the following thermal protocol: 10 min at 25°C, 2 h at 37°C, 5 min for 85°C and for 4°C. The qPCR was performed with SYBR Green PCR Master Mix (Applied Biosytems Inc., Foster City, CA, USA) on a Light cycler 96 system (Roche Applied Science, USA). The thermal cycling conditions were as follows: 95°C for 10 min and 40 cycles at 95°C for 15 s and 60°C for 1 min. The corresponding primers were showed on electronic supplementary material, table S3. Each qPCR was repeated at least three times to achieve the best reproducibility data. GAPDH was used as an endogenous control to normalize gene expression data. Amplification of the genes of interest and GAPDH was performed simultaneously. Relative mRNA expression was determined based on the cycle threshold (CT) and calculated using the equation 2^−ΔΔCT^: ΔCT_treatment_ = CT_target_ − CT_GAPDH_; ΔCT_control_ = CT_target_ − CT_GAPDH_; ΔΔCT = ΔCT_treatment_ − ΔCT_control_.

#### 2.4.4. Protein extraction and western blotting

PBMCs and brain tissue lysed on ice for 30 min in RIPA lysis buffer containing phenylmethylsulfonyl fluoride and centrifuged at 12 000 r.p.m. for 15 min at 4°C; the supernatant was immediately transferred to a fresh tube on ice. Protein concentration was measured with the bicinchoninic acid protein assay kit (Beyotime Institute of Biotechnology, Shanghai, China) with a bovine serum albumin standard concentration curve and absorbance readings at 562 nm on a spectrophotometer. Equivalent amounts of protein (30 µg) were separated by 10% acrylamide sodium dodecyl sulfate-polyacrylamide gel electrophoresis and electrophoretically transferred to a polyvinylidenedifluoride membrane that was blocked with 5% nonfat milk and probed with primary antibodies against CB1R, CB2R, NAPE-PLD, FAAH, DAGL-α, MAGL and GAPDH. The membrane was then incubated with horseradish peroxidase-conjugated goat anti-rabbit, and goat anti-mouse secondary antibody (antibodies details seen in electronic supplementary material, table S4). Protein bands were detected with an enhanced chemiluminescence western blotting detection kit (Beyotime Institute of Biotechnology, Shanghai, China). Results were analysed using Quantity One software (Bio-Rad Laboratories, Hercules, CA, USA) to obtain the optical density ratio of the target protein to GAPDH. Measurements were obtained for triplicate samples.

### 2.5. Statistical analyses

The results were presented as means ± s.d. or means ± s.e.m. which were analysed using GraphPad Prism 7.0 (GraphPad software, CA, USA). The comparison of the data was analysed by carrying out a one-way analysis of variance (ANOVA) or paired Student's *t-*test. A repeated-measures ANOVA was conducted to evaluate the differences in escape latency in the Morris water maze test. Dunnett's *post hoc* test was applied for multiple comparison (comparing all groups to VPA group). All reported *p* values were two-tailed, and the statistical significance was set at the *α* = 0.05 level.

## 3. Results

### 3.1. Comparison of the components of the eCB system between cases and controls

**Figure 1.**
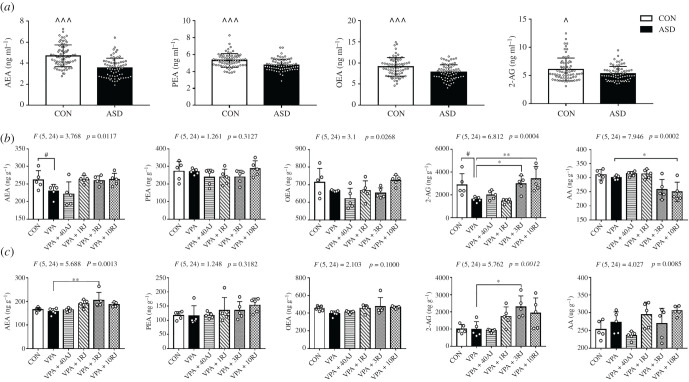
The levels of endogenous cannabinoids. (*a*) The plasma levels of AEA, PEA, OEA and 2-AG in the participants (*n* = 70 children per group). Effects of JZL184 treatment on the levels of AEA, PEA, OEA, 2-AG and AA in hippocampus (*b*) and in the PFC (*c*) in VPA-exposed offspring (*n* = 5 pups per group). The error bars represent s.d. (*a*) Results were analysed by paired Student's *t-*test (^*p* < 0.05, ^^^*p* < 0.001). (*b*,*c*) Results were analysed by one-way analysis of variance with Dunnett's *post hoc* test (^#^*p* < 0.05, versus CON group; *p < 0.05, **p < 0.01, versus VPA group). AJ, acute JZL184 treatment; RJ, repeated JZL184 treatment.

Autistic children had lower plasma concentrations of AEA, PEA, OEA and 2-AG than healthy controls (*p* < 0.05; figure 1*a*; electronic supplementary material, table S5). The levels of PEA in the ASD group were negatively correlated with the total scores of the ABC (*r* = −0.326, *p* = 0.013, data not shown). However, only AEA and 2-AG levels in the hippocampus were significantly reduced in VPA-exposed rats compared to controls (*p* < 0.05; figure 1*b*), and there was no significant difference in the AEA, PEA, OEA and 2-AG levels in the PFC (figure 1*c*).

**Figure 2.**
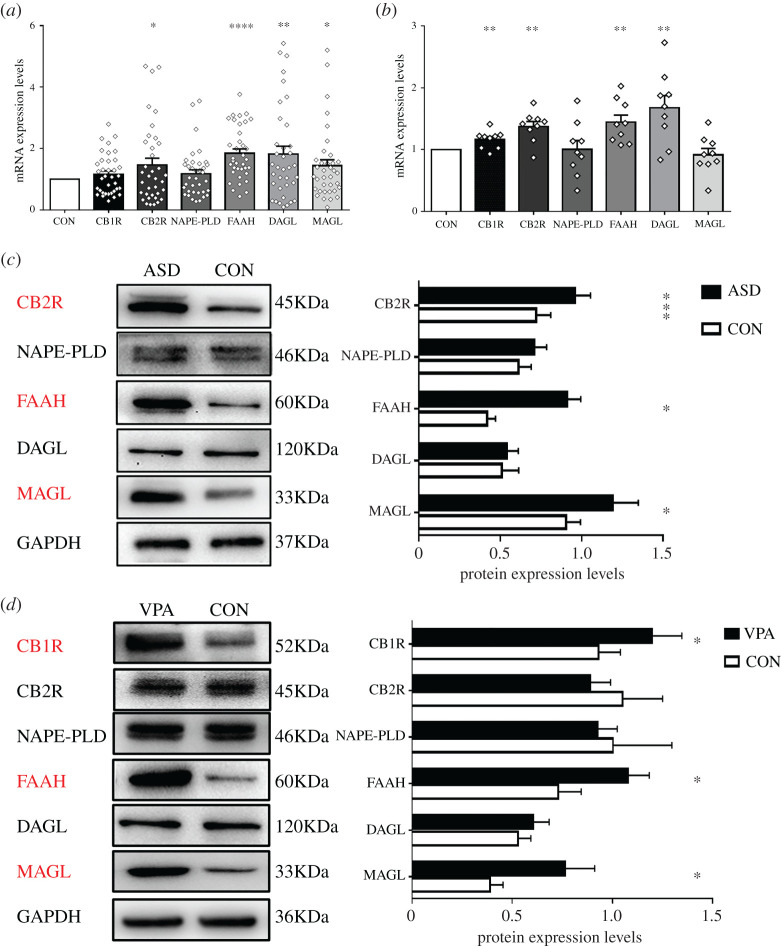
The components of eCB system expression levels in cases and controls. (*a*) Relative mRNA expression levels normalized to GAPDH in PBMCs of participants (*n* = 36 children per group). (*b*) Relative mRNA expression levels normalized to GAPDH in hippocampus of rats (*n* = 9 pups per group). (*c*) Relative protein expression levels normalized to GAPDH in the PBMCs of participants (*n* = 27 children per group); while CB1R protein expression was not detected. (*d*) Relative protein expression levels normalized to GAPDH in the hippocampus of rats (*n* = 9 pups per group). Specificities of monoclonal antibodies (except for CB1R: polyclonal antibody). Data represented as means ± s.e.m. *p < 0.05, **p < 0.01, ***p < 0.001, ****p < 0.0001 from paired student's *t*-test.

CB2R, FAAH, DAGL and MAGL mRNA levels in PBMCs from autistic children were significantly higher than those from the healthy controls, and CB2R, FAAH and MAGL protein levels in PBMCs were also higher (*p* < 0.05; figure 2*a*,*c*). However, CB1R protein expression was not detected. Due to no significant differences in eCB levels in the PFC, we then solely investigated hippocampal eCB system expression. Compared with controls, rats prenatally exposed to VPA exhibited differences in mRNA expression of CB1R, CB2R, FAAH and DAGL in the hippocampus (*p* < 0.05). There was a significant increase in CB1R, FAAH and MAGL protein levels, whereas CB2R and DAGL protein levels did not reach the significance level (figure 2*b*,*d*).

### 3.2. Effect of JZL184 treatment on autism-like phenotypes

**Figure 3.**
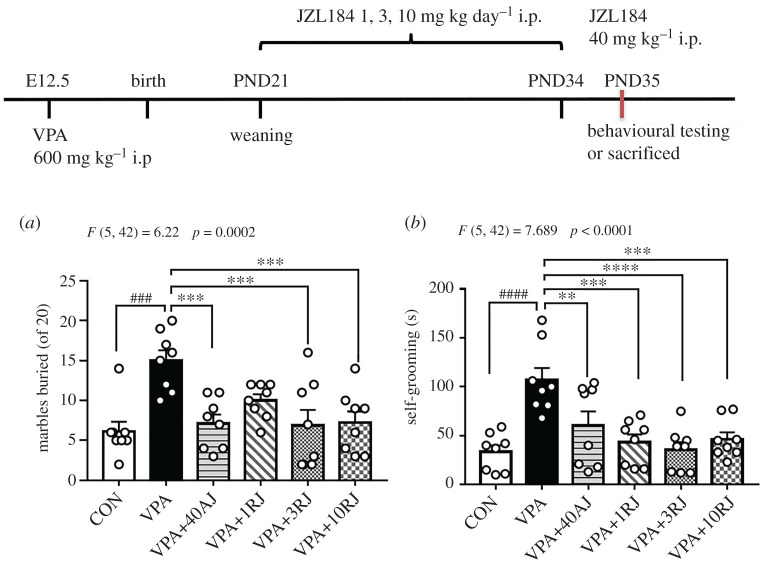
Flowchart of JZL184 administration in VPA-exposed offspring and effects of JZL184 treatment on repetitive and stereotypical behaviors of VPA-exposed offspring in marble burying test and self-grooming test. (*a*) The number of marbles buried. (*b*) The self-grooming time. Data represented as means ± s.e.m. (*n* = 8 pups per group). Results were analysed by one-way ANOVA with Dunnett's *post hoc* test (^###^*p* < 0.001, ^####^*p* < 0.0001, versus CON group; **p < 0.01, ***p < 0.001, ****p < 0.0001 versus VPA group). AJ, acute JZL184 treatment; RJ, repeated JZL184 treatment.

One-way ANOVA analyses revealed statistically significant result between the groups with respect to marbles buried (*F*_5,42_ = 6.22, *p* = 0.0002). VPA-exposed rats buried more marbles than control rats (*p* = 0.0001), and JZL184-treated rats buried significantly less marbles than VPA-exposed rats (VPA versus VPA + 40AJ: *p* = 0.0007, VPA versus VPA + 3RJ: *p* = 0.0005, VPA versus VPA + 10RJ: *p* = 0.0009; figure 3*a*). A significant difference was found with respect to repetitive self-grooming behaviour (*F*_5,42_ = 7.689, *p* < 0.0001). The *post hoc* test confirmed that VPA-exposed rats spent more time self-grooming than control rats (*p* < 0.0001), and self-grooming behaviour was significantly reduced by JZL184 treatment (VPA versus VPA + 40AJ: *p* = 0.0087, VPA versus VPA + 1RJ: *p* = 0.0002, VPA versus VPA + 3RJ: *p* = 0.0001, VPA versus VPA + 10RJ: *p* = 0.0005; figure 3*b*). All in all, JZL184 administration could improve the repetitive and stereotypical behaviours of VPA-exposed rats.

**Figure 4.**
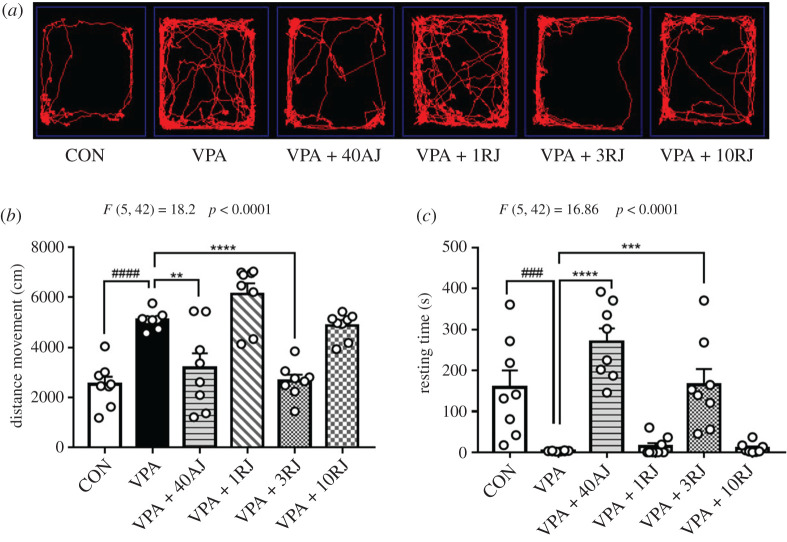
Effects of JZL184 treatment on locomotor activity of VPA-exposed offspring in open field test. (*a*) Representative images of locomotor tracks. (*b*) Distance movement. (*c*) Resting time. Data represented as means ± s.e.m. (*n* = 8 pups per group). Results were analysed by one-way ANOVA with Dunnett's *post hoc* test (^###^*p* < 0.001, ^####^*p* < 0.0001, versus CON group; **p < 0.01, ***p < 0.001, ****p < 0.001 versus VPA group). AJ, acute JZL184 treatment; RJ, repeated JZL184 treatment.

We logged the distance moved and resting time to further gauge anxiety-like behaviour. One-way ANOVA analyses revealed statistically significant result in the vase of locomotor activity among the groups (distance moved: *F*_5,42_ = 18.2, *p* < 0.0001, resting time: *F*_5,42_ = 16.86, *p* < 0.0001). Upon exposure to a novel brightly lit aversive open field arena, VPA-exposed rats exhibited hyperlocomotion as demonstrated by an increase in distance moved and a decrease in resting time when compared to the control rats (*p* < 0.0001, *p* = 0.0010, respectively; figure 4*a–c*). JZL184 acute injection and 3 mg kg^−1^ repeated injections could reverse the elevated locomotor activity of VPA-exposed rats (distance moved: *p* = 0.0017, *p* = 0.0001, respectively; resting time: *p* < 0.0001, *p* = 0.0006, respectively; figure 4*b*,*c*). By contrast, 1 and 10 mg kg^−1^ repeated injections failed to exhibit this effect.

**Figure 5.**
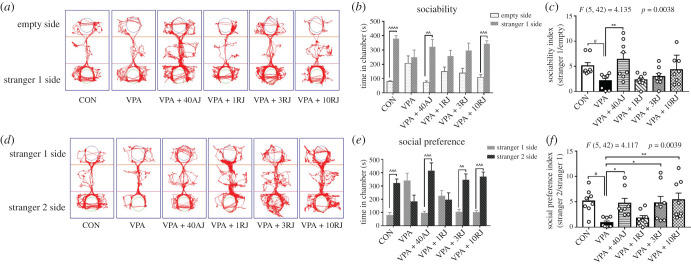
Effects of JZL184 treatment on social behaviors of VPA-exposed offspring in three-chamber test. (*a*–*c*) Sociability test. (*d*,*e*) Social preference test. (*a*,*d*) Representative images of track movements. (*b*) Time spent in chamber of sociability towards stranger rat 1 or empty cage. (*c*) Sociability index, calculated as time spent in the stranger 1/empty cage. (*e*) Time spent in chamber of social preference towards stranger 1 or stranger 2. (*f*) Social preference index, was calculated as time spent in the stranger 2/the stranger 1. Data represented as means ± s.e.m. (*n* = 8 pups per group). (*b*,*e*) Results were analysed by paired Student's *t*-test (^^*p* < 0.01, ^^^*p* < 0.001, ^^^^*p* < 0.001, stranger 1 side versus empty side or stranger 2 side). (*c*,*f*) Results were analysed by one-way ANOVA with Dunnett's *post hoc* test (^#^*p* < 0.05 versus CON group; *p < 0.05, **p < 0.01 versus VPA group). AJ, acute JZL184 treatment; RJ, repeated JZL184 treatment.

Prenatal VPA exposure impaired social interaction behaviours in offspring as reported previously [20,21]. In the habituation phase, rats from all of the experimental groups did not show any significant chamber preference (data not shown). In the sociability test, VPA-exposed groups exhibited the typical ASD-like phenotype and failed to demonstrate a significant preference for the unfamiliar rat compared with the object. Acute administration of JZL184 and 10 mg kg^−1^ administered by repeated injections corrected this aberrant behaviour (*p* = 0.0017, *p* = 0.0006, respectively; figure 5*a*,*b*). However, only acute administration significantly enhanced the sociability index (*p* = 0.0035; figure 5*c*). In the social preference test, which determines whether the experimental rats show a preference for a socially novel rat or familiar one, VPA-exposed rats exhibited reduced social novelty recognition compared to the control rats, which was quantified by the time spent engaging in investigatory behaviour with ‘stranger rat 2’ and the social preference index (*p* = 0.0006, *p* = 0.0132, respectively; figure 5*d*–*f*). Acute administration of JZL184, and repeated injections at doses of 3 and 10 mg kg^−1^ increased the amount of time spent by ‘stranger 2’ (*p* = 0.0009, *p* = 0.0034, *p* = 0.0004, respectively, figure 5*e*) and the social preference index (*p* = 0.0297, *p* = 0.0258, *p* = 0.0077, respectively; figure 5*f*). By contrast, the influence of 1 mg kg^−1^ JZL184 repeated injection on social behaviours could not be observed. These results suggested that JZL184 administration could restore impaired social interaction.

**Figure 6.**
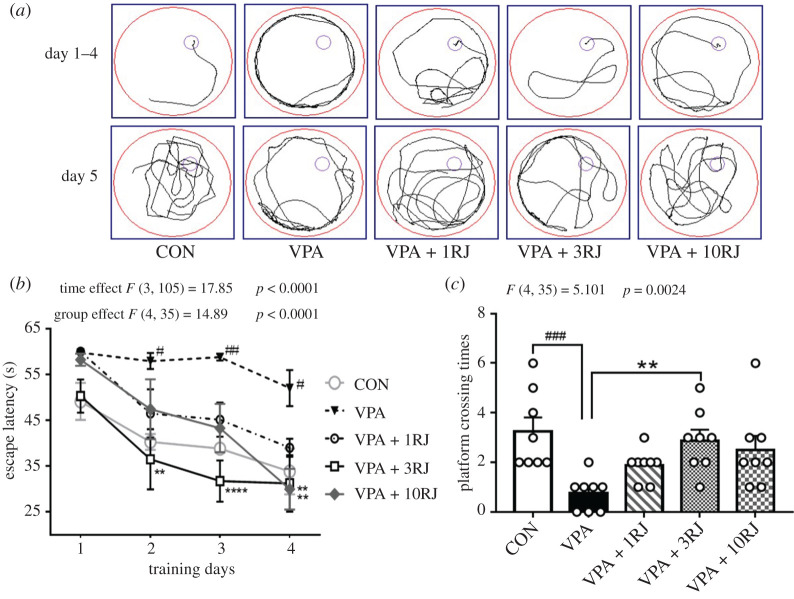
Effects of JZL184 treatment on learning and memory of VPA-exposed offspring in Morris water maze test. (*a*) Representative images of the escape latency and the passing times through the platform area. (*b*) Escape latency on different training days. (*c*) Times of rats passing the site where there had been a platform. Data represented as means ± s.e.m. (*n* = 8 pups per group). (*b*) Results were analysed by repeated-measures ANOVA. (*c*) Results were analysed by one-way ANOVA with Dunnett's *post hoc* test (^#^*p* < 0.05, ^##^*p* < 0.01, ^###^*p* < 0.001 versus CON group; **p < 0.01, ****p < 0.001 versus VPA group). RJ, repeated JZL184 treatment.

In the Morris water maze test, the rats are required to find a hidden platform in order to escape from swimming in a pool of water. During the training trial, a decrease was observed in the escape latency in all of the groups, and the differences were observed among groups during the same day (repeated-measures ANOVA: group effect: *F*_4,35_ = 14.89, *p* < 0.0001; time effect: *F*_3,105_ = 17.85, *p* < 0.0001; interaction effect between group and time: *F*_12,105_ = 0.93, *p* = 0.52; figure 6*a*,*b*). A *post hoc* test showed that compared with the control group, VPA-exposed rats required a longer escape latency, whereas repeated treatment with 3 or 10 mg kg^−1^ of JZL184 notably shortened the escape latency during the training period (2nd, 3rd and 4th day, *p* < 0.05; figure 6*a*,*b*). On the 5th day, one-way ANOVA analyses revealed statistically significant differences between the groups in the spatial probe test (*F*_4,35_ = 5.101, *p* = 0.0024; figure 6*c*). The results indicated VPA-exposed rats were not able to remember the original platform, and the number of crossings of the former platform location (passing time) was less than that observed in the control rats (*p* = 0.0010). Interestingly, VPA-exposed rats administrated with JZL184 repeated injections at doses of 3 mg kg^−1^ significantly increased platform crossing times (*p* = 0.0052; figure 3*a*,*c*). Taken together, JZL184 3 mg kg^−1^ repeated treatment could improve learning and spatial memory deficits in VPA-induced rats.

### 3.3. Effect of JZL184 treatment on the components of the eCB system

Acute administration of JZL184 (40 mg kg^−1^) did not alter eCB levels in the hippocampus and the PFC (figure 1*b*,*c*). Repeated administration of JZL184 at doses of 3 and 10 mg kg^−1^ enhanced the levels of 2-AG in the hippocampus and corresponding reductions in its metabolite, AA. There were no differences in the levels of AEA, PEA and OEA (figure 1*b*). Repeated administration of JZL184 at doses of 3 mg kg^−1^ increased the levels of AEA and 2-AG in the PFC without affecting the levels of PEA, OEA and AA (figure 1*c*).

**Figure 7.**
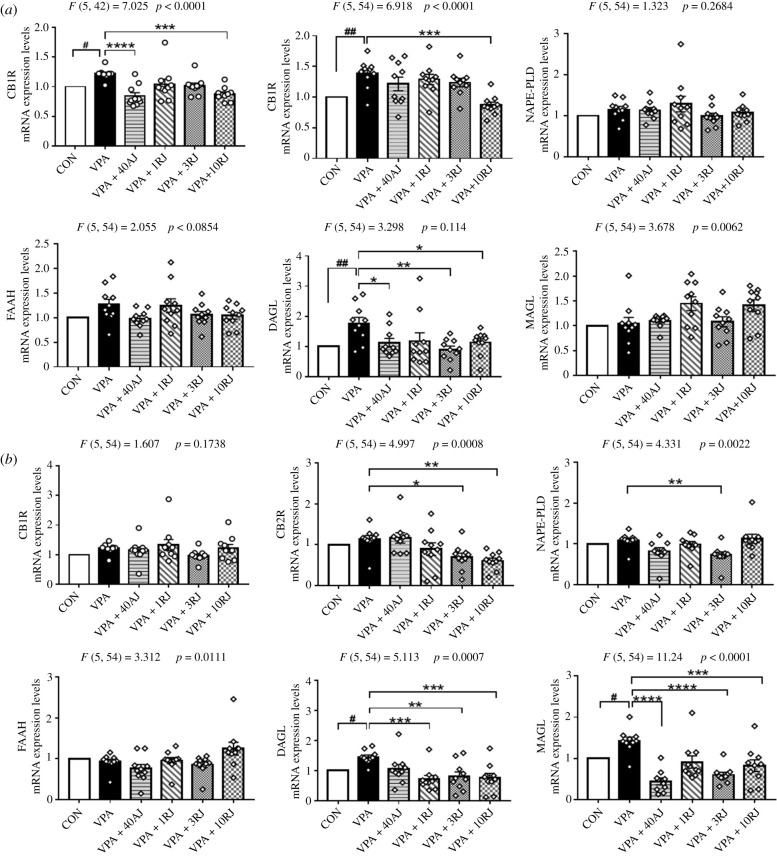
Effects of JZL184 treatment on the relative mRNA expression levels of eCB system normalized to GAPDH in hippocampus (*a*) and in the PFC (*b*). Data represented as means ± s.e.m. (*n* = 10 pups per group). Results were analysed by one-way ANOVA with Dunnett's *post hoc* test (^#^*p* < 0.05, ^##^*p* < 0.01 versus CON group; *p < 0.05, **p < 0.01, ***p < 0.001, ****p < 0.001 versus VPA group). AJ, acute JZL184 treatment; RJ, repeated JZL184 treatment.

As shown in figure 7*a*, CB1R, CB2R and DAGL mRNA expression in the hippocampus were lower in VPA-exposed rats than those in control rats (*p* < 0.05), which was consistent with prior results (figure 2*b*). Acute administration of JZL184 (40 mg kg^−1^) reduced CB1R and DAGL mRNA levels of VPA-exposed offspring in the hippocampus (*p* < 0.05). Repeated injections of JZL184 at doses of 3 mg kg^−1^ influenced DAGL mRNA expression (*p* = 0.0026). Repeated injections of JZL184 at doses of 10 mg kg^−1^ decreased CB1R, CB2R and DAGL mRNA expression (*p* = 0.0001, *p* = 0.0001, *p* = 0.0396, respectively).

As shown in figure 7*b*, DAGL and MAGL mRNA expression in the PFC were increased in VPA-exposed rats when compared to controls rats (*p* = 0.0454, *p* = 0.0195, respectively), while there were not significant differences in CB1R, CB2R, NAPE-PLD, FAAH mRNA expression. Acute administration of JZL184 (40 mg kg^−1^) reduced MAGL mRNA levels of VPA-exposed offspring (*p* = 0.0001). JZL184 1, 3 and 10 mg kg^−1^ repeated injections influenced CB2R, NAPE-PLD, DAGL and MAGL mRNA levels in the PFC (*p* < 0.05).

## 4. Discussion

This study aimed to highlight that ASD children and ASD model rats have been found to exhibit disruption of the eCB system, and that pharmacological modulators of the eCB system may offer therapeutic potential in ASD. Our results of reduced eCB content, increased degradation of enzymes and compensatory upregulation of CBRs suggested lower eCB signalling in ASD. Moreover, we observed that JZL184 treatment, by enhancing intrinsic 2-AG levels, ameliorated autistic behaviours in VPA-exposed offspring. This finding was characterized by reduced repetitive and stereotypical behaviours in marble burying and self-grooming test, reduced hyperactivity in the open field test, increased sociability and social preference in the three-chamber test and improved cognitive functioning in the Morris water maze test. This research is important to encourage the identification of potential targets for improved therapeutic treatments in ASD.

To date, only two studies with humans provided evidence regarding eCB levels in blood samples of ASD children [6,7], and findings of which demonstrated lower concentrations of AEA, PEA and OEA in autistic children, which are consistent with the findings of the present study. Notably, this is the first time that we found lower circulating 2-AG levels in children with ASD. We also found that children with ASD who have lower PEA levels exhibited more serious ASD symptoms. Interestingly, Kelly *et al.* [24] demonstrated that children with poor communication scores at age 3 years had been found to exhibit dysregulated plasma eCB levels at 1 year of age, which implicated that eCBs may be a potential biomarker for the early diagnosis of ASD. Anecdotally, case reports from Italy first corroborated that PEA, whether alone or combination with other natural supplements, can refine ASD core impairments [25]. Subsequently, an Iranian randomized, double-blind placebo-controlled trial revealed that PEA (600 mg administered twice daily) may augment the therapeutic effects of risperidone on ASD-related irritability and hyperactivity without serious side effects [26]. The non-psychoactive, medical cannabis (e.g. cannabidiol, cannabidivarin) in ASD patients appears to be well tolerated and safe (low side effects rate), and may be an effective treatment in some countries to relieve autistic symptoms [27,28]. Nevertheless, cannabinoid treatment remains a controversial ethical issue with respect to individuals with ASD, and any kind of cannabinoid consumption is illegal in China. Collectively, whether as a diagnostic biomarker or as a potential therapeutic target, decreased eCB levels were associated with ASD.

Confirming the results found in human studies, we observed that the levels of two major eCBs, namely, AEA and 2-AG, were reduced in the hippocampus of VPA-induced rats. However, Kerr *et al.* [11] found that the levels of AEA, PEA, OEA and 2-AG in the hippocampus did not differ between VPA-exposed and control rats. Intriguingly, they demonstrated that eCB levels were enhanced in the hippocampus of VPA-exposed rats immediately following the sociability test, i.e. eCB content was susceptible to behavioural testing, which is supported by many other studies [29,30]. To this end, in the present study, the behavioural experiments were paralleled by biochemical measurement, whereas Kerr examined eCB concentration 72 h after the animals underwent behavioural experiments. This might account for the disparity in the two studies. In addition, several lines of work pointed out that eCB changes appeared to be region-specific, so we did not observe a similar profile and magnitude of eCBs in the hippocampus and in the PFC. In addition, changes in the eCB system are more pronounced in the hippocampus with respect to the PFC [13,31].

In the light of PBMCs (i.e. lymphocytes, NK cells and monocytes) which could serve as a tool to investigate eCB system changes in the CNS in several neuropsychiatric disorders, we also examined eCB-associated receptors and enzymes in PBMCs isolated from the whole blood of autistic children. CB1R is the most abundant G protein-coupled receptor located in the brain, while CB2R is sparsely expressed in the brain and is instead primarily present in immunoregulatory cells, such as microglia and peripheral immune cells [32]. This could explain that the differences in CB1R mRNA expression in PBMCs and CB2R protein expression in the hippocampus were not observed between ASD cases and controls; even CB1R protein expression was not detected in PBMCs in the present study. It is noteworthy that we found that transcription and translation of CB2R in PBMCs and CB1R in the hippocampus were all enhanced. Siniscalco *et al.* [8] found results that are similar to those of the present study, which revealed unchanged CB1R mRNA levels and upregulated CB2R mRNA and protein levels in ASD-PBMCs in comparison to healthy subjects. The Zamberletti study also found that prenatal VPA exposure increased CB1R protein levels [13]. Remarkably, CB1R and CB2R activation exerted diverse consequences across cellular physiology. As the main molecular target of eCBs, CB1R is found high density on presynaptic termini of glutamatergic and GABAergic neurons, and activation of CB1R is implicated in the regulation of excitatory-inhibitory balance, synaptic strength and neurotransmitter release, which ultimately mediates social functioning, learning and memory [33,34]. Furthermore, genetic variants in *CNR1* (encoding CB1R) gene were correlated not only to verbal but to non-verbal social communication in ASD research studies [35,36]. Additionally, via activation of CB2R, the eCB system exerts anti-inflammatory actions and decreases glial activity to prevent excessive inflammation and cell damage [32,37]. We could not obtain brain tissue of patients. Nonetheless, to some extent, the expression of CB2R on peripheral immune cells reflects changes in the CNS [38]. In fact, this study also found an increase in mRNA expression of CB2R in the hippocampus of VPA-exposed offspring. Hence, our findings supported the notion that the eCB system plays a protective role in inflammatory responses in autistic children via increasing CB2R expression [8]. However, further experimental evidence towards neuroinflammation is needed. Because it is impossible that the changes in the components of the eCB system are independent from one another, researchers suggested that there might be negative feedback regulation of eCBs and CBR densities [39,40]. We deduced that upregulation of CBRs reflected a response to the lower levels of eCBs observed in autistic patients and model rats in the current study. Alterations in CBRs levels are transient adaptive reactions which attempt to restore normal homeostasis that is otherwise disrupted by the disease.

Thus far, only Siniscalco's team have previously investigated the eCB system in PBMCs from individuals with ASD, but they incorporated neither 2-AG metabolic enzymes nor protein expression of eCB-related enzymes into their study [8,9]. To our knowledge, this is the first study to explore relatively entire components of the eCB system in PBMCs from humans. The biosynthesis of 2-AG can be catalyzed by two DAGL isoforms, namely DAGL-α and DAGL-β. DAGL-α, which are expressed throughout the brain, and 2-AG levels dropped by up to 80–90% in the brain in the DAGL-α null mouse brain [41]. Given that DAGL-α is the main 2-AG synthesizing enzyme in the brain, this study only examined DAGL-α expression (DAGL for short). Surprisingly, we revealed that DAGL mRNA expression was increased in the human and rat sample, but not protein. Moreover, we found concurrent increases in the expression of FAAH and MAGL, which are responsible for AEA and 2-AG degradation, both in PBMCs and in the hippocampus. The lower eCB levels in the present study may account for the increased degradation of enzymes. The contradictory results that both DAGL and MAGL mRNA expression were increased in the PFC may explain the unchanged levels of eCBs in the PFC. Interestingly, in Siniscalco's study, FAAH mRNA expression did not significantly change, and NAPE-PLD slightly decreased in 17 cases of ASD and 22 cases of healthy controls [8]. Our findings from animals were in keeping with previous reports of upregulated expression of FAAH and MAGL in VPA-induced rats [13,42]. As a whole, our results and those of other studies highlight the presence of decreased eCB signalling in ASD children and in the animal model which might explain the deficits exhibited in the cognitive and social domains.

The animal model studies have shown that prenatal VPA exposure in rodents recapitulates ASD-like pathophysiology at a molecular, cellular and behavioural level. VPA-induced rats have been developed and became a widely used environmental preclinical model of ASD with strong face and construct validity, which also serves as a good platform for testing pharmacological reagents that might be used to treat ASD. On a behavioural level, our findings have confirmed that VPA-exposed rats exhibit the core symptoms of ASD, impaired social interaction and repetitive behaviour, and possibly co-occurring emotional and cognitive problems. Furthermore, we found that prenatal VPA exposure induces a disturbance of the eCB system in offspring rats that is similar that observed in ASD children, which is in accordance with prior research studies [11,13], indicating a reduced eCB tone in ASD. Therefore, we evaluated the efficacy of boosting 2-AG levels by administering the hydrolysis inhibitor JZL184 which attenuates repetitive and stereotypical behaviours, hyperactivity, and deficits in social and cognitive functioning in VPA-induced rats. The results showed that either acute or chronic administration of JZL184 was successful in mitigating ASD-like behaviours, which was in line with previous reports involving *Fmr1* knockout mice and *Shank3B*^−/−^ mice [10,18,19].

Rats treated with acute administration 40 mg kg^−1^ JZL184 exhibited reduced repetitive marble burying, grooming behaviours and hyperactivity, as well as an improvement in sociability and social preference induced by VPA exposure. We observed that a single injection of JZL184 (40 mg kg^−1^) did not change the levels of eCBs in the hippocampus and PFC, which was consistent with the findings of Kerr *et al.* [43] who did not detect an alternation in the levels of eCBs 2.5 h following injection of JZL184 (10 mg kg^−1^). Nonetheless, JZL184 at a dose of 40 mg kg^−1^ could show loss of MAGL activity [44]. Kruk-Slomka *et al.* [45] demonstrated similar results, which revealed that acute injection of JZL184 40 mg kg^−1^ significantly decreased locomotion and improved long-term acquisition of memory and learning processes. Although only decreased CB1R and DAGL mRNA expression in the hippocampus and decreased MAGL mRNA expression in the PFC were detected and the eCB levels were not acute JZL184 treatment still exerted a positive effect on behaviours. Repeated treatment with JZL184 at a dose of 3 mg kg^−1^ had a restorative effect on repetitive marble-burying and grooming behaviours, locomotor activity, social preference, learning and spatial memory. By contrast, a high dose (10 mg kg^−1^) only partially affected repetitive behaviours, social preference and learning, and a low dose which was tested (1 mg kg^−1^) was ineffective. In fact, the behavioural efficacious dose of 3 mg kg^−1^ robustly increased 2-AG levels in both the hippocampus and PFC, whereas concomitantly, a marginal increase in AEA levels was observed. In this regard, *Dagla*^−/−^ animals showed an extensive reduction in 2-AG levels and a concomitant decrease in AEA in the hippocampus and cortex. Furthermore, administration of JZL184 to *Dagla*^−/−^ mice increased not only 2-AG levels but also the level of AEA [41]. Schlosburg *et al.* [17] also confirmed that chronic dosing also caused a modest elevation in AEA. These data, together with our similar results, suggested a crosstalk of 2-AG and AEA production in the brain; however, the underlying mechanism is not known. We should note that cumulative exposure to JZL184 probably generates a partial effect of blockade on FAAH, rather than MAGL contributing directly to the degradation of AEA [17]. Our findings also indicated that the increase of 2-AG in the hippocampus and PFC might not result from the increase of DAGL, because JZL184 treatment reduced DAGL mRNA expression. Inversely, the increase of 2-AG exerted an inhibitory effect on the DAGL expression. Convergent literature demonstrated that sustained elevation of 2-AG in the brain, caused by either genetic deletion or chronic pharmacological blockade of MAGL, led to CB1R desensitization and tolerance to CB1R agonists, as well as to significant decreases in CB1R number and function, and this effect would limit the therapeutic potential of JZL184 [17]. A chronic JZL184 dose of 16 mg kg^−1^ daily (typically one week) reliably produced tolerance [46]. The current study observed that a dose of 10 mg kg^−1^ daily (two weeks) reduced CB1R and CB2R expression in the hippocampus, and CB2R expression in the PFC. Thus, repeated administration of a low dose of JZL184 (i.e. 3 mg kg^−1^ in the current study) could produce elevated eCB brain levels without behavioural tolerance and CBRs desensitization. Additionally, acute treatment with JZL184 also has an important effect on CBRs [22]. We hypothesized that JZL184 treatment could improve ASD-like behaviours via CBR-dependent and -independent mechanisms: (i) as 2-AG acts via CB1R and generally suppresses synaptic transmission, neuronal excitability and neurogenesis, the improvement of eCB-induced synaptic plasticity could ameliorate ASD-like behaviours [10,18]; and (ii) 2-AG is an important metabolic intermediate in lipid synthesis and it also serves as a major source of AA, which is required for pro-inflammatory prostaglandin synthesis. Pharmacological inactivation of MAGL induced not only elevations in 2-AG, but reductions in the product AA and downstream AA-derived eicosanoids as well. This impairment of eicosanoids production is a direct consequence of the reduction in AA rather than the augmentation of eCB signalling, which is possibly relevant to cyclooxygenase enzymes [44]. Furthermore, inactivation of MAGL could suppress the pro-inflammatory cytokines production and microglial activation induced by LPS [44,47]. Eventually, independent of CBRs, increasing 2-AG provides protection against neuroinflammation, which then ameliorates ASD-like behaviours.

This study had some limitations that must be taken into account in interpretating the results. First, the present study is limited by the use of male offspring only. The eCB system is known to exhibit sexual dimorphism in humans and rodents, particularly in CB1R expression and functionality [31,48]. Second, the study could have benefited from comparisons with plasma eCB levels in VPA-induced rats. Third, this study may lack some information of value due to not having evaluated enzyme and receptor activity, and additional non-cannabinoid receptor targets which are known to have affinity and activity to eCBs. While the therapeutic use of the eCB system is inviting, extensive research is required to further evaluate this complex regulatory pathway and the safety of pharmacological manipulation.

Current evidence strongly implicates alterations in the eCB system in human patients with ASD and in animal models. The reduced eCB content, elevated degradation of enzymes and compensatory upregulation of CBRs indicates reduced eCB signalling in ASD. In addition, augmentation of 2-AG levels by pharmacological inhibition of MAGL resulted in the normalization of ASD-related behavioural abnormalities in VPA-exposed offspring. The improvement of behavioural phenotypes was consistent with the observed increase in 2-AG in the hippocampus and PFC following administration of JZL184 at a dose of 3 mg kg^−1^. These data provide preclinical evidence which supports the ability of JZL184 to ameliorate behavioural abnormalities resembling core and associated symptoms of ASD. The high heterogeneity in the phenotypic presentation of ASD poses investigative and clinical challenges for treatment, and subgroups of ASD individuals may benefit more from drugs that increase cannabinoid levels.

## Abbreviations

2-AG, 2-arachidonoylglycerol; AA, arachidonic acid; ABC, Autism Behaviour Checklist; ADI-R, Autism Diagnostic Interview-Revised; ADOS, Autism Diagnostic Observation Schedule; AEA, anandamide; ANOVA, one-way analysis of variance; ASD, autism spectrum disorder; BMI, body mass index; CARS, Childhood Autism Rating Scales; CB1R, type 1 cannabinoid receptors; CB2R, type 2 cannabinoid receptors; CNS, central nervous system; CSS, calibrated severity scores; DAGL, diacylglycerol lipase; eCB, endocannabinoid; FAAH, fatty acid amide hydrolase; LC-MS/MS, liquid chromatography-tandem mass spectrometry; MAGL, monoacylglycerol lipase; NAPE-PLD, *N*-acylphosphatidylethanolamine-specific phospholipase D; OEA, oleoylethanolamide; PBMC, peripheral blood mononuclear cell; PEA, palmitoylethanolamide; PND, postnatal day; qPCR, quantitative PCR; SRS, Social Responsiveness Scale; VABS, Vineland Adaptive Behaviour Scale; VPA, valproic acid.

## Data accessibility

The dataset used and/or analysed during the current study are available from the corresponding author on reasonable request.

## References

[1] American Psychiatric Association. 2013 Diagnostic and statistical manual of mental disorders, pp. 58-62. 5th edn. Washington, DC: American Psychiatric Association.

[2] Sun X et al. 2019 Autism prevalence in China is comparable to Western prevalence. Mol. Autism **10**, 7. (10.1186/s13229-018-0246-0) 30858963 PMC6394100

[3] Maenner MJ et al. 2020 Prevalence of autism spectrum disorder among children aged 8 years: autism and developmental disabilities monitoring network, 11 sites, United States, 2016. Morb. Mortal. Wkly Rep. Surveill. Summ. **69**, 1-12. (10.15585/mmwr.ss6904a1) PMC7119644 32214087

[4] Blankman JL, Simon GM, Cravatt BF. 2007 A comprehensive profile of brain enzymes that hydrolyze the endocannabinoid 2-arachidonoylglycerol. Chem. Biol. **14**, 1347-1356. (10.1016/j.chembiol.2007.11.006) 18096503 PMC2692834

[5] Zou MY, Li DX, Li L, Wu L, Sun C. 2019 Role of the endocannabinoid system in neurological disorders. Int. J. Dev. Neurosci. **76**, 95-102. (10.1016/j.ijdevneu.2019.03.002) 30858029

[6] Karhson DS, Krasinska KM, Dallaire JA, Libove RA, Phillips JM, Chien AS, Garner JP, Hardan AY, Parker KJ. 2018 Plasma anandamide concentrations are lower in children with autism spectrum disorder. Mol. Autism **9**, 18. (10.1186/s13229-018-0203-y) 29564080 PMC5848550

[7] Aran A et al. 2019 Lower circulating endocannabinoid levels in children with autism spectrum disorder. Mol. Autism **10**, 2. (10.1186/s13229-019-0256-6) 30728928 PMC6354384

[8] Siniscalco D et al. 2013 Cannabinoid receptor type 2, but not type 1, is up-regulated in peripheral blood mononuclear cells of children affected by autistic disorders. J. Autism Dev. Disord. **43**, 2686-2695. (10.1007/s10803-013-1824-9) 23585028

[9] Siniscalco D, Bradstreet JJ, Cirillo A, Antonucci N. 2014 The in vitro GcMAF effects on endocannabinoid system transcriptionomics, receptor formation, and cell activity of autism-derived macrophages. J. Neuroinflammation **11**, 78. (10.1186/1742-2094-11-78) 24739187 PMC3996516

[10] Jung KM et al. 2012 Uncoupling of the endocannabinoid signalling complex in a mouse model of fragile X syndrome. Nat. Commun. **3**, 1080. (10.1038/ncomms2045) 23011134 PMC3657999

[11] Kerr DM, Downey L, Conboy M, Finn DP, Roche M. 2013 Alterations in the endocannabinoid system in the rat valproic acid model of autism. Behav. Brain Res. **249**, 124-132. (10.1016/j.bbr.2013.04.043) 23643692

[12] Melancia F, Schiavi S, Servadio M, Cartocci V, Campolongo P, Palmery M, Pallottini V, Trezza V. 2018 Sex-specific autistic endophenotypes induced by prenatal exposure to valproic acid involve anandamide signalling. Br. J. Pharmacol. **175**, 3699-3712. (10.1111/bph.14435) 29968249 PMC6109221

[13] Zamberletti E, Gabaglio M, Woolley-Roberts M, Bingham S, Rubino T, Parolaro D. 2019 Cannabidivarin treatment ameliorates autism-like behaviors and restores hippocampal endocannabinoid system and glia alterations induced by prenatal valproic acid exposure in rats. Front. Cell Neurosci. **13**, 367. (10.3389/fncel.2019.00367) 31447649 PMC6696797

[14] Gould GG, Burke TF, Osorio MD, Smolik CM, Zhang WQ, Onaivi ES, Gu T-T, Desilva MN, Hensler JG. 2014 Enhanced novelty-induced corticosterone spike and upregulated serotonin 5-HT1A and cannabinoid CB1 receptors in adolescent BTBR mice. Psychoneuroendocrinology **39**, 158-169. (10.1016/j.psyneuen.2013.09.003) 24126181 PMC3893037

[15] Wei D, Dinh D, Lee D, Li D, Anguren A, Moreno-Sanz G, Gall CM, Piomelli D. 2016 Enhancement of anandamide-mediated endocannabinoid signaling corrects autism-related social impairment. Cannabis Cannabinoid Res. **1**, 81-89. (10.1089/can.2015.0008) 28861483 PMC5549436

[16] Wu H-F, Lu T-Y, Chu M-C, Chen PS, Lee C-W, Lin H-C. 2020 Targeting the inhibition of fatty acid amide hydrolase ameliorate the endocannabinoid-mediated synaptic dysfunction in a valproic acid-induced rat model of autism. Neuropharmacology **162**, 107736. (10.1016/j.neuropharm.2019.107736) 31398381

[17] Schlosburg JE et al. 2010 Chronic monoacylglycerol lipase blockade causes functional antagonism of the endocannabinoid system. Nat. Neurosci. **13**, 1113-1119. (10.1038/nn.2616) 20729846 PMC2928870

[18] Wang W et al. 2018 Treating a novel plasticity defect rescues episodic memory in Fragile X model mice. Mol. Psychiatry **23**, 1798-1806. (10.1038/mp.2017.221) 29133950 PMC5951717

[19] Folkes OM et al. 2020 An endocannabinoid-regulated basolateral amygdala-nucleus accumbens circuit modulates sociability. J. Clin. Investig. **130**, 1728-1742. (10.1172/JCI131752) 31874107 PMC7108917

[20] Wu H, Wang X, Gao J, Liang S, Hao Y, Sun C, Xia W, Cao Y, Wu L. 2017 Fingolimod (FTY720) attenuates social deficits, learning and memory impairments, neuronal loss and neuroinflammation in the rat model of autism. Life Sci. **173**, 43-54. (10.1016/j.lfs.2017.01.012) 28161158

[21] Wu H, Zhang Q, Gao J, Sun C, Wang J, Xia W, Cao Y, Hao Y, Wu L. 2018 Modulation of sphingosine 1-phosphate (S1P) attenuates spatial learning and memory impairments in the valproic acid rat model of autism. Psychopharmacology **235**, 873-886. (10.1007/s00213-017-4805-4) 29218394

[22] Sugaya Y, Yamazaki M, Uchigashima M, Kobayashi K, Watanabe M, Sakimura K, Kano M. 2016 Crucial roles of the endocannabinoid 2-arachidonoylglycerol in the suppression of epileptic seizures. Cell Rep. **16**, 1405-1415. (10.1016/j.celrep.2016.06.083) 27452464

[23] Gobira PH et al. 2019 Opposing roles of CB(1) and CB(2) cannabinoid receptors in the stimulant and rewarding effects of cocaine. Br. J. Pharmacol. **176**, 1541-1551. (10.1111/bph.14473) 30101419 PMC6487550

[24] Kelly RS et al. 2019 Metabolomics and communication skills development in children; evidence from the ages and stages questionnaire. Metabolites **9**, 42. (10.3390/metabo9030042) 30841573 PMC6468693

[25] Antonucci N, Cirillo A, Siniscalco D. 2015 Beneficial effects of palmitoylethanolamide on expressive language, cognition, and behaviors in autism: a report of two cases. Case Rep. Psychiatry **2015**, 325061. (10.1155/2015/325061) 26491593 PMC4602323

[26] Khalaj M et al. 2018 Palmitoylethanolamide as adjunctive therapy for autism: efficacy and safety results from a randomized controlled trial. J. Psychiatr. Res. **103**, 104-111. (10.1016/j.jpsychires.2018.04.022) 29807317

[27] Bar-Lev Schleider L, Mechoulam R, Saban N, Meiri G, Novack V. 2019 Real life experience of medical cannabis treatment in autism: analysis of safety and efficacy. Sci. Rep. **9**, 200. (10.1038/s41598-018-37570-y) 30655581 PMC6336869

[28] Aran A, Cassuto H, Lubotzky A, Wattad N, Hazan E. 2019 Brief report: cannabidiol-rich cannabis in children with autism spectrum disorder and severe behavioral problems—a retrospective feasibility study. J. Autism Dev. Disord. **49**, 1284-1288. (10.1007/s10803-018-3808-2) 30382443

[29] Gould GG et al. 2012 Acetaminophen differentially enhances social behavior and cortical cannabinoid levels in inbred mice. Prog. Neuropsychopharmacol. Biol. Psychiatry **38**, 260-269. (10.1016/j.pnpbp.2012.04.011) 22542870 PMC3389197

[30] Wei D, Lee D, Cox CD, Karsten CA, Peñagarikano O, Geschwind DH, Gall CM, Piomelli D. 2015 Endocannabinoid signaling mediates oxytocin-driven social reward. Proc. Natl. Acad. Sci. USA **112**, 14 084-14 089. (10.1073/pnas.1509795112) 26504214 PMC4653148

[31] Craft RM, Marusich JA, Wiley JL. 2013 Sex differences in cannabinoid pharmacology: a reflection of differences in the endocannabinoid system? Life Sci. **92**, 476-481. (10.1016/j.lfs.2012.06.009) 22728714 PMC3492530

[32] Araujo DJ, Tjoa K, Saijo K. 2019 The endocannabinoid system as a window into microglial biology and its relationship to autism. Front. Cell Neurosci. **13**, 424. (10.3389/fncel.2019.00424) 31619967 PMC6759510

[33] Karhson DS, Hardan AY, Parker KJ. 2016 Endocannabinoid signaling in social functioning: an RDoC perspective. Transl. Psychiatry **6**, e905. (10.1038/tp.2016.169) 27676446 PMC5048207

[34] Osborne AL, Solowij N, Babic I, Lum JS, Newell KA, Huang X-F, Weston-Green K. 2019 Effect of cannabidiol on endocannabinoid, glutamatergic and GABAergic signalling markers in male offspring of a maternal immune activation (poly I:C) model relevant to schizophrenia. Prog. Neuropsychopharmacol. Biol. Psychiatry **95**, 109666. (10.1016/j.pnpbp.2019.109666) 31202911

[35] Smith DR, Stanley CM, Foss T, Boles RG, Mckernan K. 2017 Rare genetic variants in the endocannabinoid system genes CNR1 and DAGLA are associated with neurological phenotypes in humans. PLoS ONE **12**, e0187926. (10.1371/journal.pone.0187926) 29145497 PMC5690672

[36] Chakrabarti B, Baron-Cohen S. 2011 Variation in the human cannabinoid receptor CNR1 gene modulates gaze duration for happy faces. Mol. Autism **2**, 10. (10.1186/2040-2392-2-10) 21714860 PMC3155489

[37] Mecha M et al. 2018 2-AG limits Theiler's virus induced acute neuroinflammation by modulating microglia and promoting MDSCs. Glia **66**, 1447-1463. (10.1002/glia.23317) 29484707

[38] Centonze D, Battistini L, Maccarrone M. 2008 The endocannabinoid system in peripheral lymphocytes as a mirror of neuroinflammatory diseases. Curr. Pharm. Des. **14**, 2370-2382. (10.2174/138161208785740018) 18781987

[39] Neumeister A et al. 2013 Elevated brain cannabinoid CB1 receptor availability in post-traumatic stress disorder: a positron emission tomography study. Mol. Psychiatry **18**, 1034-1040. (10.1038/mp.2013.61) 23670490 PMC3752332

[40] Doenni VM, Gray JM, Song CM, Patel S, Hill MN, Pittman QJ. 2016 Deficient adolescent social behavior following early-life inflammation is ameliorated by augmentation of anandamide signaling. Brain Behav. Immun. **58**, 237-247. (10.1016/j.bbi.2016.07.152) 27453335 PMC5461973

[41] Jenniches I et al. 2016 Anxiety, stress, and fear response in mice with reduced endocannabinoid levels. Biol. Psychiatry **79**, 858-868. (10.1016/j.biopsych.2015.03.033) 25981172

[42] Servadio M et al. 2016 Targeting anandamide metabolism rescues core and associated autistic-like symptoms in rats prenatally exposed to valproic acid. Transl. Psychiatry **6**, e902. (10.1038/tp.2016.182) 27676443 PMC5048215

[43] Kerr DM, Harhen B, Okine BN, Egan LJ, Finn DP, Roche M. 2013 The monoacylglycerol lipase inhibitor JZL184 attenuates LPS-induced increases in cytokine expression in the rat frontal cortex and plasma: differential mechanisms of action. Br. J. Pharmacol. **169**, 808-819. (10.1111/j.1476-5381.2012.02237.x) 23043675 PMC3687661

[44] Nomura DK et al. 2011 Endocannabinoid hydrolysis generates brain prostaglandins that promote neuroinflammation. Science **334**, 809-813. (10.1126/science.1209200) 22021672 PMC3249428

[45] Kruk-Slomka M, Banaszkiewicz I, Slomka T, Biala G. 2019 Effects of fatty acid amide hydrolase inhibitors acute administration on the positive and cognitive symptoms of schizophrenia in mice. Mol. Neurobiol. **56**, 7251-7266. (10.1007/s12035-019-1596-0) 31004320 PMC6815283

[46] Murataeva N, Straiker A, Mackie K. 2014 Parsing the players: 2-arachidonoylglycerol synthesis and degradation in the CNS. Br. J. Pharmacol. **171**, 1379-1391. (10.1111/bph.12411) 24102242 PMC3954479

[47] Szafran BN, Lee JH, Borazjani A, Morrison P, Zimmerman G, Andrzejewski K, Ross M, Kaplan B. 2018 Characterization of endocannabinoid-metabolizing enzymes in human peripheral blood mononuclear cells under inflammatory conditions. Molecules **23**, 3167. (10.3390/molecules23123167) 30513753 PMC6321211

[48] Rubino T, Parolaro D. 2011 Sexually dimorphic effects of cannabinoid compounds on emotion and cognition. Front. Behav. Neurosci. **5**, 64. (10.3389/fnbeh.2011.00064) 21991251 PMC3181427

